# Expansion of deciduous tall shrubs but not evergreen dwarf shrubs inhibited by reindeer in Scandes mountain range

**DOI:** 10.1111/1365-2745.12753

**Published:** 2017-03-16

**Authors:** Tage Vowles, Bengt Gunnarsson, Ulf Molau, Thomas Hickler, Leif Klemedtsson, Robert G. Björk

**Affiliations:** ^1^ Department of Biological and Environmental Sciences University of Gothenburg Box 461 40530 Göteborg Sweden; ^2^ Senckenberg Biodiversity & Climate Research Centre Bik F Senckenberganalge 25 D‐60325 Frankfurt Germany; ^3^ Department of Physical Geography Goethe University Frankfurt Altenhöferallee 1 D‐60438 Frankfurt Germany; ^4^ Department of Earth Sciences University of Gothenburg Box 460 40530 Göteborg Sweden

**Keywords:** *Betula nana*, *Calluna vulgaris*, *Empetrum nigrum*, mountain birch forest, plant diversity, plant–herbivore interactions, reindeer, shrub heath

## Abstract

1. One of the most palpable effects of warming in Arctic ecosystems is shrub expansion above the tree line. However, previous studies have found that reindeer can influence plant community responses to warming and inhibit shrubification of the tundra.
2. We revisited grazed (ambient) and ungrazed study plots (exclosures), at the southern as well as the northern limits of the Swedish alpine region, to study long‐term grazing effects and vegetation changes in response to increasing temperatures between 1995 and 2011, in two vegetation types (shrub heath and mountain birch forest).
3. In the field layer at the shrub heath sites, evergreen dwarf shrubs had increased in cover from 26% to 49% but were unaffected by grazing. Deciduous dwarf and tall shrubs also showed significant, though smaller, increases over time. At the birch forest sites, the increase was similar for evergreen dwarf shrubs (20–48%) but deciduous tall shrubs did not show the same consistent increase over time as in the shrub heath.
4. The cover and height of the shrub layer were significantly greater in exclosures at the shrub heath sites, but no significant treatment effects were found on species richness or diversity.
5. July soil temperatures and growing season thawing degree days (TDD) were higher in exclosures at all but one site, and there was a significant negative correlation between mean shrub layer height and soil TDD at the shrub heath sites.
6. *Synthesis*. This study shows that shrub expansion is occurring rapidly in the Scandes mountain range, both above and below the tree line. Tall, deciduous shrubs had benefitted significantly from grazing exclosure, both in terms of cover and height, which in turn lowered summer soil temperatures. However, the overriding vegetation shift across our sites was the striking increase in evergreen dwarf shrubs, which were not influenced by grazing. As the effects of an increase in evergreen dwarf shrubs and more recalcitrant plant litter may to some degree counteract some of the effects of an increase in deciduous tall shrubs, herbivore influence on shrub interactions is potentially of great importance for shaping arctic shrub expansion and its associated ecosystem effects.

## Introduction

In recent years, several studies have noted a shift in plant abundance and community structure in alpine and arctic areas, many of them linking these shifts to increasing temperatures (ACIA 2005; Elmendorf *et al*. 2012b; Gottfried *et al*. 2012). An increase in above‐ground biomass has been seen above the tree line (Tape, Sturm & Racine 2006; Myers‐Smith *et al*. 2011), as well as below it (Tømmervik *et al*. 2004; Hedenås *et al*. 2011), over the past decades. The expansion of tall shrubs, such as birch (*Betula* spp.) and willow (*Salix* spp.), perhaps constitutes the most striking change in tundra vegetation composition, and has been observed in many alpine and arctic ecosystems (e.g. Sturm, Racine & Tape 2001; Tape, Sturm & Racine 2006; Myers‐Smith *et al*. 2011; Naito & Cairns 2011; Cramer *et al*. 2014; Myers‐Smith *et al*. 2015).

Above the tree line, tall shrubs are usually the largest plant life‐form and can influence biodiversity, albedo, snow cover, nutrient availability and soil temperature (Myers‐Smith *et al*. 2011). The increased canopy height and density of shrubs on the tundra cause an increase in the absorption of incoming radiation and a decrease in albedo compared to shrub‐free tundra (Chapin *et al*. 2005; Sturm *et al*. 2005). Higher canopies also trap more snow which leads to higher soil temperatures during the winter (Sturm *et al*. 2001; Myers‐Smith & Hik 2013). In the summer, on the other hand, shading from canopies can decrease soil temperatures and active layer depths (Blok *et al*. 2010). These changes in soil temperature, along with increases in litter input, may in turn have implications for nutrient cycling. Larger nitrogen pools and faster mineralization rates during the summer have been found in tall compared to low dwarf birch vegetation due to input of higher quality litter (Buckeridge *et al*. 2010) and the increased winter temperatures beneath shrubs have been hypothesized to increase annual mineralization rates by 25% (Chapin *et al*. 2005), possibly leading to positive feedback effects on primary production. Furthermore, shading from denser shrub canopies can have negative impacts on tundra species richness (Cornelissen *et al*. 2001; Walker *et al*. 2006; Pajunen, Oksanen & Virtanen 2011). Consequently, an increase in shrub biomass could have fundamental effects on tundra ecosystems, by dramatically changing living conditions for a range of species as well as potentially influencing carbon and nitrogen cycles.

Herbivores exert top‐down effects and play an important part in shaping subarctic and alpine plant communities (e.g.Manseau, Huot & Crete 1996; van der Wal *et al*. 2007; Post & Pedersen 2008; Speed *et al*. 2012; Olofsson, te Beest & Ericson 2013; Austrheim *et al*. 2014; Speed *et al*. 2014; Christie *et al*. 2015). In northern Scandinavia, reindeer *Rangifer tarandus* can impact plant community composition through grazing, trampling and fertilization (Sørensen *et al*. 2009). During the winter, reindeer diets consist mainly of lichens and evergreen shrubs, while graminoids and more palatable deciduous shrubs become more important as spring progresses (Bergerud 1972; Skogland 1984; Ophof, Oldeboer & Kumpula 2013). Several studies have found that reindeer can influence plant community responses to warming and inhibit shrub expansion (Post & Pedersen 2008; Olofsson *et al*. 2009; Ravolainen *et al*. 2011; Cahoon *et al*. 2012; Kaarlejärvi, Hoset & Olofsson 2015), as well as impede tree line advancement (Cairns & Moen 2004; van Bogaert *et al*. 2011). Reindeer can also influence nitrogen and carbon dynamics; either directly, through deposition of faeces (Stark, Strommer & Tuomi 2002; Barthelemy, Stark & Olofsson 2015), or indirectly, through altering species composition and allocation patterns (Olofsson, Stark & Oksanen 2004; Stark, Julkunen‐Tiitto & Kumpula 2007; Cahoon *et al*. 2012; Lindwall *et al*. 2013), and through trampling, which can increase soil temperatures (van der Wal, van Lieshout & Loonen 2001; Olofsson 2009).

It is likely that herbivore influence on species composition would, in turn, also have an effect on plant diversity, and there is some evidence that reindeer grazing reduces species richness in low‐productive habitats (Austrheim & Eriksson 2001; Eskelinen & Oksanen 2006; Moen & Lagerström 2008). On the other hand, as an expanding shrub cover can decrease understorey species richness (Pajunen, Oksanen & Virtanen 2011; Pajunen, Virtanen & Roininen 2012; Post 2013), by holding back shrub expansion, the net effect of grazing on species richness may be positive. Generalizations about the effects of herbivory on diversity are made difficult by the many influencing factors, such as elevation (Speed, Austrheim & Mysterud 2013), vegetation type (Pajunen, Virtanen & Roininen 2012), evolutionary history and the available pool of colonizers (Moen & Danell 2003), but it is evident that our knowledge about the processes that shape these dynamics is limited.

Large‐scale studies have observed a general trend of thermophilization of arctic and alpine plant communities (Gottfried *et al*. 2012; Elmendorf *et al*. 2015) but there is substantial variation in the change seen in different plant groups at a more local level (e.g. Wilson & Nilsson 2009; Hedenås *et al*. 2012). The impact of grazing, too, has been assigned varying degrees of importance (Moen & Danell 2003). Furthermore, in Scandinavia, most studies on reindeer grazing and vegetation change have been carried out in the northern part of Norway or Finland or in the region around Abisko in Sweden (Bernes *et al*. 2015). These studies come predominantly from tundra sites, while data from subalpine forest are rarer (Bernes *et al*. 2015). Thus, despite an increasing body of research on grazer impact in arctic and alpine areas, there is still an abiding need to experimentally test the impact of herbivory across differing gradients in climate and vegetation throughout alpine Scandinavia.

We studied the effect of the past two decades of warming on vegetation composition in the Fennoscandian mountain range by resurveying 16‐year‐old permanently marked grazed and ungrazed plots. Specifically, we wanted to investigate whether the increase in shrub cover observed round the Arctic extends to the southern as well as to the more frequently studied northern limit of the Swedish alpine range (Bernes *et al*. 2015), above and below the tree line. We hypothesize that shrub increase will be greater and plant diversity lower in the absence of grazing. We expect this effect to be larger in deciduous than in evergreen shrubs, and larger in the summer pastures of the shrub heath than the birch forest sites, due to deciduous shrubs being a preferred source of food to reindeer during the summer (Bergerud 1972). In turn, an increased shrub cover will cause summer soil temperatures to be lower in exclosures than in reindeer‐trampled ambient plots, and winter temperatures to be higher, due to increased shading and snow trapping respectively.

## Materials and methods

### Study areas

**Figure 1:**
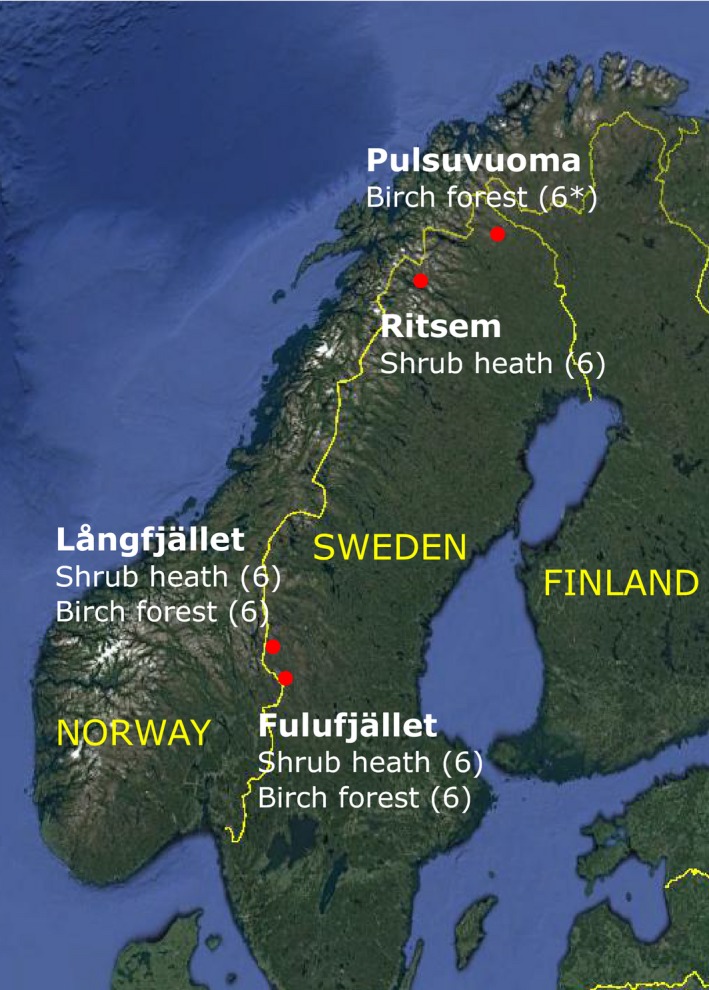
Google earth map of Sweden showing the six study sites, three in each vegetation type. Numbers of plots at each site given in brackets. *At Pulsuvuoma, only two of the original ambient plots could be found, so a new one was laid out (but was not used in analyses of time effects). [Colour figure can be viewed at wileyonlinelibrary.com]

The study was carried out in four locations along the Swedish mountain range, two in the southern part and two in the northern part. Two vegetation types were studied, shrub heath (above the forest line) and mountain birch forest. Both the southern areas, Fulufjället and Långfjället (61°32′N, 12°45′E and 62°05′N, 12°16′E, respectively, see Table S1, Supporting Information for coordinates for all plots), contained both a shrub heath and a mountain birch forest site, whereas the two northern areas, Ritsem and Pulsuvuoma (67°46′N, 17°32′E and 68°20′N, 21°19′E), consisted of just one vegetation type; heath in Ritsem and birch forest in Pulsuvuoma. Thus, the total number of sites was six, three in each vegetation type (Fig. 1). At all sites the bedrock is nutrient‐poor and chemically acidic (with the exception of the Ritsem site which is locally calcareous) and the soil consists of gravelly till (Eriksson, Niva & Caruso 2007).

Fulufjället and Långfjället are situated in a region of continental to sub‐continental climate (Oksanen 1998; Sjörs 1999). At Fulufjället, average annual precipitation at the closest meteorological station (Gördalen) was 834 mm between 1961 and 1990 (Swedish Meteorological and Hydrological Institute (SMHI) 2009). The heath site is situated on a north westerly facing slope at an altitude of 930 m a.s.l. and the vegetation is dominated by dwarf shrubs *Empetrum nigrum* ssp*. hermaphroditum* (Hagerup) Böcher, *Calluna vulgaris* L. Hull*, Vaccinium vitis‐idaea* L. and *Vaccinium myrtillus* L. and tall deciduous shrub *Betula nana* L., along with narrow‐leaved graminoid *Deschampsia flexuosa* L. The bottom layer consists of a thick (10–12 cm) and expansive lichen cover dominated by *Cladonia* and *Cetraria*‐species, but bryophytes of the *Dichranum* and *Polytrichum* genera are also common. The reason for the thick lichen layers is that reindeer husbandry has not been practised in Fulufjället since the 19th century (Naturvårdsverket 2002). Therefore, the Fulufjället sites served as control sites, allowing us to ensure that any effects found were actually from the exclusion of large herbivores and not from the fences themselves. The birch forest site is located about 3 km west of the heath site at 880 m a.s.l. The tree layer here consists almost entirely of *Betula pubescens* ssp*. czerepanovii* (N. I. Orlova) Hämet‐Ahti. The field layer is made up of dwarf shrubs *E. nigrum*,* V. vitis‐idaea* and *V. myrtillus* along with graminoids *D. flexuosa and Nardus stricta* L., and forbs such as *Melanpyrum pratense* L. and *Solidago virgaurea* L. The bottom layer consists of mosses such as *Pleurozium shreberi* (Brid.) Mitt. and *Dicranum* species, and of *Cladonia* and *Cetraria* lichens. At Långfjället, the heath site lies on an easterly slope at 840 m a.s.l. The birch forest site is roughly 5 km to the southwest at 800 m a.s.l. The mean annual precipitation between 1961 and 1990 was 697 mm (at Grövelsjön, SMHI 2009). The flora at Långfjället is very similar to Fulufjället, with the most noticeable difference being that the lichens do not form thick carpets like they do at the Fulufjället heath, and that the tree layer at the birch forest site includes scattered occurrences of Scots pine, *Pinus sylvestris* L. At the Långfjället heath, reindeer graze the site from approximately June–September, whereas the birch forest site is grazed in spring and autumn, as the reindeer migrate to summer pastures from the winter pastures and back again (J. Jonsson, Idre Sami village, pers. comm. 2014).

The most northerly site of Pulsuvuoma is also situated in a region of continental to sub‐continental climate (Oksanen 1998; Sjörs 1999) but is more strongly continental than the southern sites with a lower annual precipitation (444 mm at Övre Soppero 1961–1990; SMHI 2009). It is also at a considerably lower elevation (460 m a.s.l.). The vegetation, however, is much the same as in the birch forests at the southern sites. The Pulsuvuoma site is frequented by reindeer approximately between November and January, although this varies considerably between years (P‐G idivuoma, Lainiovuoma Sami village, pers. comm. 2015). The Ritsem site is further west and is more influenced by westerly winds, resulting in a more oceanic climate (Eriksson, Niva & Caruso 2007), with a mean annual precipitation 1961–1990 of 510 mm (SMHI 2009). The site is located on a south‐easterly slope at an altitude of 800 m a.s.l. and, in contrast to the other sites, where the underlying bedrock is chemically acidic, the bedrock at Ritsem is made up of mica schist that is soft, relatively easily weathered and locally calcareous, which has an influence on the vegetation (Eriksson, Niva & Caruso 2007). The area is more species‐rich than the other shrub heath sites, with more species of graminoids and forbs, for instance *Calamagrostis lapponica* (Wahlenb.) Hartm., *Carex bigelowii* Torr. ex Schwein., *Bistorta vivipara* (L.) Gray and *Hieracium* L. sect. *Alpina* (Griseb.) Gremli. In the bottom layer, *Stereocaulon* species dominate along with *Cladonia* and *Cetraria* lichens. The site at Ritsem is grazed intensely from April to December (K‐Å Pittsa, Unna Tjerusj Sami village, pers. comm. 2015).

### Experimental design

At each site, there are six 25 × 25 m plots, three of which are surrounded by a 1·7‐m high wire mesh fence to keep out herbivores (‘exclosures’). The fences keep out large herbivores like reindeer and elk, but not hares or rodents. The other three plots (from here on referred to as ‘ambient’ plots, as they represent natural, i.e. grazed, conditions) are marked only with dug out corners and are accessible to grazers. The fences were constructed in 1995 when the WWF initiated a project with the aim of studying effects of reindeer grazing and reindeer husbandry practices on the mountain vegetation in Sweden (for full background see Eriksson, Niva & Caruso 2007). Vegetation inventories were carried out in all plots prior to the erection of the fences and again 2–4 years later (Ritsem in 1997, Långfjället in 1998 and Fulufjället and Pulsuvuoma, which was named Tavvavuoma in the original study, in 1999). In 2011, we revisited the sites and following the original WWF methodology, we resurveyed the plots in order to study long time changes in vegetation composition.

The vegetation inventories were for the most part carried out during July–August 2011, but due to time constraints and difficulties with finding some of the original ambient plots, four plots at Fulufjället birch forest and one plot at Långfjället birch forest could not be surveyed until July 2012. At Pulsuvuoma, only two of the three original ambient plots could be found, so a new one was marked out.

To summarize, in all there were six sites (three heath and three birch forest) with six plots at each site (three ambient and three exclosures), inventoried on three occasions between 1995 and 2012, making 108 plot inventories in total.

### Vegetation classification

The vegetation data were divided into five broad groups according to growth form; shrubs, graminoids, forbs, lichens and mosses. Plant functional types (growth forms) have been found to be a useful framework for predicting vegetation responses to, and effects on, the environment (Chapin *et al*. 1996). We divided the shrub group into evergreen and deciduous shrubs, as deciduous shrubs are generally more palatable and preferred as food to herbivores such as reindeer (Christie *et al*. 2015). We further divided the shrub groups into three height classes, because some of the most important ecosystem effects of shrubs such as snow trapping and changes in albedo, are directly linked to canopy height (Sturm *et al*. 2001). Species were grouped according to their maximum potential height as described in Mossberg & Stenberg (2008), into three height classes (prostrate dwarf shrubs <15 cm, semi‐prostrate dwarf shrubs 15–50 cm and tall shrubs >50 cm) following Elmendorf *et al*. (2012a). Semi‐prostrate dwarf shrubs will from here on be referred to only as dwarf shrubs.

### Field layer

In each of the plots, twenty 1 × 1 m subplots were randomly chosen and the cover of each species in the subplots was visually estimated. Folding rulers were laid out along the edges of the subplots to aid visual estimation. As plants tend to stretch over each other at different heights, the estimation of the total cover (including bare ground) in the subplots could add up to more than 100%. To avoid edge effects, a 1·5 m wide strip along the edges of the plot was left out and all the subplots were selected within a 22 × 22 m net area.

The height of the tallest individual of each species in the field layer (excluding lichens and mosses) was measured in each subplot. Mean height per functional group and plot was calculated as the mean of the tallest individual from each functional group and subplot (actually giving the ‘mean tallest height’). As we did not manage to establish exactly how the original height measurements were carried out, direct comparisons of how plant heights may have changed over time were deemed unreliable. Instead we used only the 2011 data to look for exclosure effects.

### Shrub layer

As an additional estimation of the shrub layer at the heath sites, the plots were further divided into sixteen 5·5 × 5·5 m plots. We randomly selected six of these and recorded height and two perpendicular measurements of canopy diameter of all shrubs and tree saplings higher than 30 cm. Shrub area was approximated by calculating the area of each shrub as if it were a circle, using the mean of the canopy measurements as the radius.

### Diversity measurements

In order to study the effects of grazing on species assemblage and richness, three diversity measures were calculated: the reciprocal forms of Simpson's *D* and the Berger–Parker Index, and species richness. Simpson's reciprocal index is weighted towards the most abundant species in the sample and is less sensitive to species richness and was calculated according to Magurran (2004). The Berger–Parker index is a dominance measure and simply expresses the proportional abundance of the most abundant species. In order to express a higher diversity with an increase in the value of the index, the reciprocal form was used according to Magurran (2004). Species richness was calculated as the number of species found in total in the twenty 1 × 1 m subplots of each plot.

Diversity comparisons between the years were only made for the field layer (i.e. only vascular plants were included in the diversity calculations), because of suspected inconsistencies in the level of accuracy of lichen and moss species determination between the inventories. To look for differences between treatments in the 2011 data, Simpson's index, the Berger–Parker index and species richness calculations were based on the full species set and included the new ambient plot in Pulsuvuoma.

### Temperature data

Temperature loggers were placed in the centre of each plot to measure soil temperature (Tinytag plus 2 TGP‐4020; Gemini Data Loggers, Chichester, UK) at 2 cm depth. At each site, an additional logger was placed at 2 m height to measure site temperature and relative humidity (Tinytag plus 2 TGP‐4500; Gemini Data Loggers). All loggers made hourly measurements, which were used to calculate daily means. The loggers were installed in May/June 2011 at all sites except Ritsem, where loggers could not be installed until July 2013 (although site temperature was measured since August 2011). To look for treatment effects on soil temperature, e.g. through increased shading or snow trapping, mean July and January temperatures were used as a proxy for summer and winter temperatures. Due to malfunctioning loggers in some plots and differing installation dates between sites, it was not possible to get complete records for January and July soil temperatures from all sites the same year. Therefore, mean July temperatures are from 2012 for all sites except Fulufjället birch forest (from 2013) and Ritsem (from 2014). Mean January temperatures are from 2013 for all sites except Ritsem (from 2014). Consequently, the soil temperature data were only used to look for treatment effects, as comparisons between sites are not meaningful.

Thawing degree days (TDD) between 15 May and 15 September were calculated from the soil temperatures according to Molau & Mølgaard (1996). TDD is the sum of all mean daily temperatures above 0 °C and have been found to be one of the dominant environmental controls on phenology in alpine areas (Molau, Nordenhall & Eriksen 2005). The loggers were not installed in time to measure temperatures for the whole vegetation season of 2011, when the vegetation inventories were carried out, so the temperatures for 2012 were used for TDD calculations at all sites except Ritsem, where TDD sums are from 2014. Therefore, as with mean temperatures, comparisons between sites are not meaningful. Due to problems with the loggers, TDD calculations for Fulufjället birch forest were not possible.

Temperature data from the closest active SMHI stations to each site for the years 1991–2014 were compared to the meteorological base period of 1961–1990 to see if mean yearly air temperatures had increased at the sites since the experiment began. Särna was the closest SMHI station to Fulufjället and Långfjället (26 and 65 km away, respectively), for Pulsuvuoma, it was Karesuando (52 km away) and for Ritsem, it was Ritsem (15 km away). Daily mean temperatures from these stations were also compared to daily means calculated from our site loggers and used to extrapolate site temperatures by means of linear regression.

### Reindeer densities

**Figure 2:**
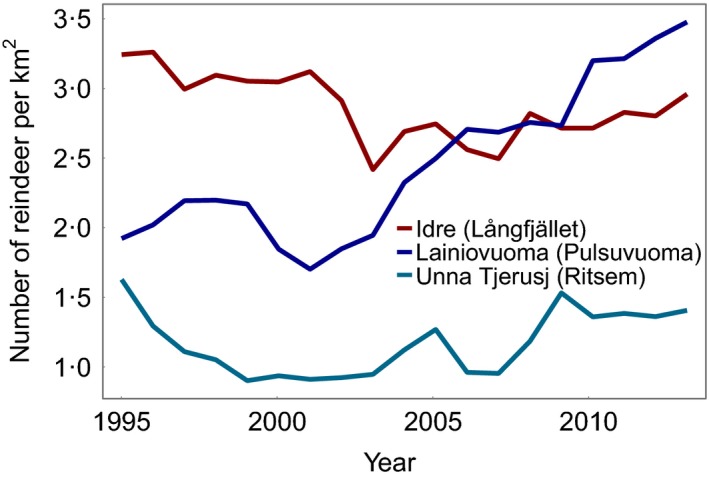
Number of reindeer per square kilometre of estimated pasture area for each Sami village encompassing our study sites. [Colour figure can be viewed at wileyonlinelibrary.com]

Reindeer numbers were obtained from the Sami Parliament. In Sweden, all reindeer are semi‐domesticated and according to The Reindeer Husbandry Act, reindeer husbandry can only be exercised by a member of a Sami village (Rennäringslag 1971:437), which reports herd sizes to the Sami Parliament every year. However, the reindeer move freely over vast areas and grazing pressure at the study sites is therefore very difficult to accurately calculate. Yet to give a rough estimate of reindeer densities at each site over the years that the plots have been in place, registered herd sizes for each Sami village in conjunction to the sites were divided by the estimated size of the total pasture area of the village (Fig. 2). At Fulufjället, there has been no reindeer husbandry since the late 19th century. On rare occasions, small groups of reindeer cross over from Norway or from Idre Sami village, but the grazing pressure, from reindeer, is considered to be negligible (Naturvårdsverket 2002). In the Results section, we therefore refer to the sites with reindeer (Långfjället heath, Ritsem, Långfjället birch forest and Pulsuvuoma) as the reindeer‐grazed sites.

### Statistical analyses

All statistical analyses were carried out with the r statistical package (R Core Team 2012). Differences in cover between years and between treatments were tested for each plant functional type using mixed effects models in library nlme (Pinheiro *et al*. 2015). We analysed birch forest and heath sites separately as they are utilized by reindeer at different times of year, when different plant groups form the main part of the diet. Time, treatment and site were treated as fixed and plot as a random factor. The coverage rate was modelled with logistic regression, as advocated by Warton & Hui (2011). However, in order to properly handle proportions equal to 0 and 1, we used a modified logit transformation, given by

$$\ln\left(\frac{\text{cov}+\delta }{1-\text{cov}+\delta }\right)$$

where cov is the mean cover of the specified plant functional type in each plot given as a proportion (bound between 0 and 1), δ = covmean × e^−3^, and covmean is the mean cover in all plots. The choice of δ should be adaptive to the typical coverage rate within each plant group, and the factor e^−3^ gives transformed zeros a few standard deviations to the left of the transformed mean. Approximate normality of the model residuals were assessed by graphical inspection of qq‐plots, and a plausible fit was seen when using the logistic transformation with δ defined as above.

To allow the effect of the treatment to change over time, separate treatment effects were used for the 2nd and 3rd inventory. As one site, Fulufjället, has no reindeer population, separate treatment effects were used for this site. The need to also include separate time effects for sites with and without reindeer population was investigated by inclusion of an interaction term between reindeer population and time in the model, using separate time effects if the interaction was significant at 5% level and using the same time effects for all sites otherwise. The lsmeans package (Lenth 2016) was used to obtain *t*‐ratios and *P*‐values for pairwise comparisons of treatment levels. Results were considered significant if *P *<* *0·05. In Pulsuvuoma, one of the ambient plots could not be found, and furthermore, because the original plot numbers had been lost, we had no possibility of knowing which plots matched which in the original data, other than if they were exclosures or ambient plots. Therefore, the Pulsuvuoma site had to be analysed separately, using a regular linear model on the transformed data. This left only two sites (Långfjället and Fulufjället) in the birch forest model.

Mixed effects models were also used to look for treatment effects on plant heights, tall shrub measurements and temperature data, where treatment and site were used as fixed factors and plot as random factor. The same model was used to look for treatment effects in diversity indices in the 2011 data, whereas effects over time were analysed using the model described for the cover estimates above. Diversity data were log transformed to fulfil assumptions of normality.

## Results

### Field layer cover estimates, shrub heath sites

**Figure 3:**
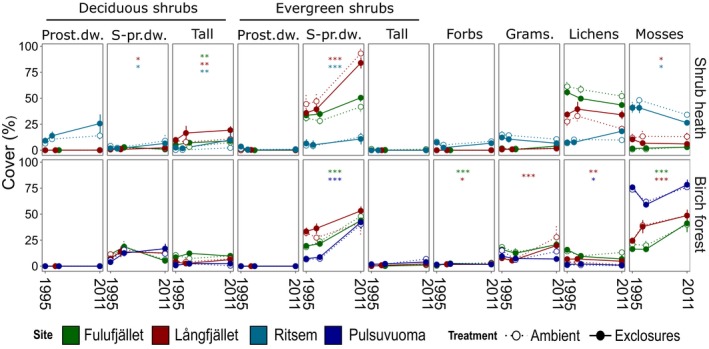
Cover change over time (mean per cent cover ± standard error) per plant functional type. Asterisks denote significant changes between 1995 and 2011 from *t*‐tests of pairwise comparisons of treatment levels (*P = 0·01–0·05, **P = 0·01–0·001, ***P < 0·001), see Table 4. Data shown are untransformed but significance levels refer to transformed data. Prost.dw., prostrate dwarf; S‐pr.dw., semi‐prostrate dwarf; grams, graminoids. [Colour figure can be viewed at wileyonlinelibrary.com]

The plant functional type that showed the largest and most consistent change over time was evergreen dwarf shrubs (Fig. 3). This group consisted primarily of the ericaceous species *E. nigrum*,* C. vulgaris* and *V. vitis‐idaea* (see Table S2 for full data on individual species cover). No significant treatment effect was found for this group (Table 1). Evergreen dwarf shrub cover had increased from a mean of 26 to 49% per square metre between 1995 and 2011 over all heath sites and treatments. Deciduous dwarf shrubs had increased significantly over time at the reindeer‐grazed sites (from 2% to 5%) and deciduous tall shrubs had increased in total at all sites (from 5% to 10%, Fig. 3 and Table 4). The deciduous dwarf group consisted first and foremost of *V. myrtillus*, while the deciduous tall group was dominated by *B. nana* and at Ritsem, *Salix glauca* L. (Table S2). Even though deciduous tall shrub cover was twice as large in exclosures as in ambient plots at reindeer‐grazed sites in 2011 (15% vs. 7%), the treatment effect was not significant. Nor was there a significant treatment effect for deciduous dwarf shrubs. Deciduous prostrate dwarf shrub cover, on the other hand, was significantly greater in exclosures than in ambient plots (13% compared to 7%), yet there was no significant time effect. This group consisted almost exclusively of *Salix herbacea* L., which was only found at Ritsem.

**Table 1:** Cover estimates (% cover of each functional group) and treatment effects from mixed effects models for the shrub heath sites. Treatment effects considered significant at *P* < 0·05 and highlighted in bold. All cover percentages given as back‐transformed values. D, deciduous; E, evergreen

Shrub heath	Cover				d.f.	Treatment effects			
	Reindeer sites (*N* = 12)		Fulufjället (*N* = 6)			Reindeer sites		Fulufjället	
	Ambient	Exclosures	Ambient	Exclosures		*t*	*P*	*t*	*P*
*1st Inventory*									
D. prostrate dwarf	4·5		0·0						
D. semiprost. dwarf	1·8		0·8						
D. tall shrubs	3·5		2·4						
E. prostrate dwarf	0·6		0·0						
E. semiprost. dwarf	27·0		31·2						
E. tall shrubs	0·0		0·5						
Forbs	3·5		0·3						
Graminoids	6·1		1·3						
Lichens	17·9		56·6						
Mosses	25·8		0·9						
*2nd Inventory*									
D. prostrate dwarf	5·2	6·8	0·0	0·1	29	−0·42	0·68	−1·07	0·29
D. semiprost. dwarf	1·8	1·3	2·3	3·0	27	0·15	0·88	0·10	0·92
D. tall shrubs	3·8	7·5	2·7	6·7	29	−0·44	0·66	0·89	0·38
E. prostrate dwarf	0·5	0·4	0·0	0·0	29	0·97	0·34		
E. semiprost. dwarf	27·0	22·7	28·2	34·8	27	0·16	0·88	−0·32	0·75
E. tall shrubs	0·0	0·0	0·5	0·0	29	0·32	0·75	−1·12	0·27
Forbs	3·1	2·0	0·2	0·3	29	2·92	**0·01**	−1·20	0·24
Graminoids	6·1	5·2	1·3	0·9	29	0·68	0·50	0·97	0·34
Lichens	20·5	20·9	60·8	49·9	29	−0·12	0·90	1·61	0·12
Mosses	29·1	22·3	1·0	2·2	27	1·64	0·11	−0·32	0·75
*3rd Inventory*									
D. prostrate dwarf	5·2	10·0	0·0	0·1	29	−2·33	**0·03**	−1·32	0·20
D. semiprost. dwarf	4·1	3·2	0·8	0·8	27	0·03	0·98	0·39	0·70
D. tall shrubs	8·1	13·6	5·9	8·5	29	−0·21	0·84	1·69	0·10
E. prostrate dwarf	0·3	0·3	0·0	0·0	29	0·54	0·59		
E. semiprost. dwarf	56·2	46·8	41·6	50·5	27	1·26	0·22	−0·40	0·70
E. tall shrubs	0·0	0·0	0·6	0·0	29	0·86	0·40	−0·84	0·41
Forbs	3·9	3·3	0·3	0·3	29	1·23	0·23	0·20	0·85
Graminoids	7·2	5·6	1·6	4·2	29	1·11	0·28	−2·70	**0·01**
Lichens	16·2	27·2	53·5	43·7	29	−3·28	**0·00**	1·42	0·17
Mosses	24·4	16·7	3·0	2·1	27	2·15	**0·04**	1·70	0·10

Lichens were significantly more abundant in exclosures at the reindeer‐grazed sites in 2011 (an increase from 19% to 26% in fenced plots and a decrease to 15% in ambient plots). Mosses changed significantly over time and between treatments at the reindeer‐grazed sites, decreasing from 26% to 24% in ambient plots and to 16% in exclosures.

**Figure 4:**
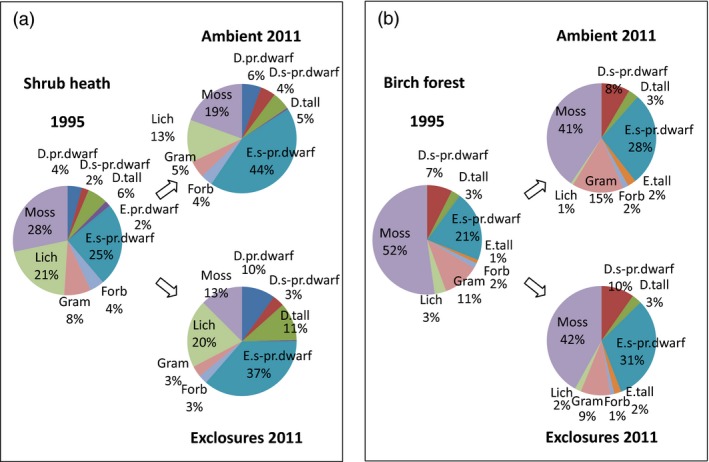
(a, b) Change in relative abundance of each plant functional type at shrub heath sites (a) and mountain birch forest sites (b). D.pr.dwarf, deciduous prostrate dwarf shrubs; D.s‐pr.dwarf, deciduous semi‐prostrate dwarf shrubs; D.tall, deciduous tall shrubs; E.pr.dwarf, evergreen prostrate dwarf shrubs, E.s‐pr.dwarf, evergreen semi‐prostrate dwarf shrubs; E.tall, evergreen tall shrubs; Forb, forbs; Gram, graminoids; Lich, lichens; Moss, mosses. [Colour figure can be viewed at wileyonlinelibrary.com]

Expressed as relative abundance, all shrub groups combined increased from 39% of total plant cover in 1995 to 59% in ambient plots and to 62% in the exclosures, but the proportional increase was larger for evergreens in ambient plots and for deciduous shrubs in exclosures. Shrubs appeared to expand mainly at the expense of mosses in exclosures, and mosses and lichens in ambient plots (Fig. 4a).

### Field layer cover estimates, mountain birch forest sites

Evergreen dwarf shrubs showed the greatest increase at the birch forest sites too, from 20% to 45% per square metre across all sites and treatments (Fig. 3, Table 4), and like at the heath sites there was no treatment effect (Tables 2 and 3). Deciduous shrub results were not as uniform and showed a lot of variation between sites. Deciduous dwarf shrubs increased at Pulsuvuoma and Långfjället (from 5% to 15% and 9% to 13% respectively), significantly so between the 1st and 2nd inventory at Pulsuvuoma. At Fulufjället, there was a significant increase from the 1st to 2nd inventory, before a significant decrease to the 3rd inventory (9% to 19% to 6%). Deciduous tall shrubs increased in total (from 5% to 6% averaged over all sites) but the effect was only significant between the second and third inventory at Långfjället and Fulufjället. At the birch forest sites, *Vaccinium uligonosum,* and not *B. nana*, was the dominant species in the deciduous tall shrub group.

**Table 2:** Cover estimates (% cover of each functional group) and treatment effects from mixed effects models for the mountain birch forest sites. Treatment effects considered significant at *P *< 0·05 and highlighted in bold. All cover percentages given as back‐transformed values. D, deciduous; E, evergreen

Birch forest	Cover				d.f.	Treatment effects			
	Reindeer sites (*N* = 6)		Fulufjället (*N* = 6)			Reindeer sites		Fulufjället	
	Ambient	Exclosures	Ambient	Exclosures		*t*	*P*	*t*	*P*
*1st Inventory*									
D. prostrate dwarf	0·0		0·0						
D. semiprost. dwarf	10·1		8·1						
D. tall shrubs	4·3		9·0						
E. prostrate dwarf	0·6		0·0						
E. semiprost. dwarf	32·8		18·7						
E. tall shrubs	0·2		0·2						
Forbs	1·6		0·7						
Graminoids	8·3		17·7						
Lichens	4·5		14·6						
Mosses	25·9		17·3						
*2nd inventory*									
D. prostrate dwarf	0·0	0·0	0·0	0·0					
D. semiprost. dwarf	13·7	12·5	18·4	18·5	15	−0·40	0·70	0·65	0·53
D. tall shrubs	2·8	2·5	6·1	12·2	17	0·00	1·00	−2·04	0·06
E. prostrate dwarf	0·5	0·4	0·0	0·0					
E. semiprost. dwarf	26·8	36·3	23·0	21·5	15	−1·72	0·11	0·49	0·63
E. tall shrubs	0·2	0·5	0·2	0·1	17	−0·95	0·36	0·58	0·57
Forbs	1·8	1·6	2·7	2·9	15	0·26	0·80	0·57	0·57
Graminoids	5·6	5·2	12·8	11·9	15	−0·01	0·99	−0·02	0·99
Lichens	3·7	6·6	9·6	9·3	15	−0·99	0·34	0·25	0·81
Mosses	33·6	37·8	23·3	16·3	17	−0·78	0·45	1·84	0·08
*3rd Inventory*									
D. prostrate dwarf	0·0	0·0	0·0	0·0					
D. semiprost. dwarf	12·4	12·0	5·8	5·1	15	−0·60	0·56	1·03	0·32
D. tall shrubs	5·5	6·2	11·5	9·2	17	−0·60	0·56	0·53	0·61
E. prostrate dwarf	0·3	0·3	0·0	0·0					
E. semiprost. dwarf	40·6	53·2	47·9	43·9	15	−1·97	0·07	0·81	0·43
E. tall shrubs	0·8	2·0	0·8	0·8	17	−1·21	0·24	0·14	0·89
Forbs	3·1	2·1	3·4	1·8	15	1·13	0·28	2·56	**0·02**
Graminoids	24·6	19·8	20·3	20·6	15	0·64	0·53	−0·30	0·77
Lichens	1·3	4·3	13·2	5·8	15	−2·08	0·06	2·60	**0·02**
Mosses	50·3	48·9	38·0	40·9	17	0·25	0·81	−0·51	0·61

**Table 3:** Cover estimates (% cover of each functional group) and treatment effects from linear models for Pulsuvuoma. Treatment effects considered significant at *P* < 0·05 and highlighted in bold. All cover percentages given as back‐transformed values. D, deciduous; E, evergreen

Pulsuvuoma	Cover (*N* = 5)		d.f.	Treatment effects	
	Ambient	Exclosures		*t*	*P*
*1st inventory*					
D. prostrate dwarf	0·0				
D. semiprost. dwarf	3·8				
D. tall shrubs	0·8				
E. prostrate dwarf	0·0				
E. semiprost. dwarf	6·6				
E. tall shrubs	1·2				
Forbs	1·7				
Graminoids	11·9				
Lichens	1·3				
Mosses	74·8				
*2nd inventory*					
D. prostrate dwarf	0·0	0·0	12		
D. semiprost. dwarf	13·9	12·0	12	0·31	0·76
D. tall shrubs	1·4	2·2	12	−0·53	0·61
E. prostrate dwarf	0·0	0·0	12		
E. semiprost. dwarf	7·0	8·6	12	−0·81	0·43
E. tall shrubs	1·9	1·6	12	0·22	0·83
Forbs	2·0	2·4	12	−0·47	0·65
Graminoids	5·5	7·2	12	−0·89	0·39
Lichens	0·9	2·0	12	−1·67	0·12
Mosses	62·0	59·2	12	0·58	0·57
*3rd inventory*					
D. prostrate dwarf	0·0	0·0	12		
D. semiprost. dwarf	11·9	15·6	12	−0·50	0·63
D. tall shrubs	0·5	1·9	12	−0·88	0·39
E. prostrate dwarf	0·0	0·0	12		
E. semiprost. dwarf	40·1	41·9	12	−0·27	0·79
E. tall shrubs	6·8	2·5	12	1·10	0·29
Forbs	2·1	1·8	12	0·32	0·76
Graminoids	14·2	6·7	12	2·37	**0·04**
Lichens	0·5	0·8	12	−0·69	0·50
Mosses	76·0	79·2	12	−0·79	0·44

Forbs had increased significantly at Fulufjället, while graminoids and lichens showed more shifting results across the sites (Tables 2, 3, 4). The most striking change was in moss cover, which increased from 19% to 41% in Fulufjället and from 24% to 49% in Långfjället.

**Table 4:** Effects over time from mixed effects model and from linear model for Pulsuvuoma (bold values signifcant at *P* < 0·05). One analysis was carried out for each vegetation type, but Pulsuvuoma had to be analysed separately using a regular linear model as original plot numbers were not known. Additionally, separate time effects for sites with and without reindeer population were used if the interaction between reindeer population and time was significant at 5% significance level

		Time	d.f.	Deciduous shrubs						Evergreen shrubs						Forbs		Graminoids		Lichens		Mosses	
				Prostrate dwarf		Semi‐prostrate dwarf		Tall		Prostrate dwarf		Semi‐prostrate dwarf		Tall									
				*t*	*P*	*t*	*P*	*t*	*P*	*t*	*P*	*t*	*P*	*t*	*P*	*t*	*P*	*t*	*P*	*t*	*P*	*t*	*P*
Shrub heath	Total	1st–3rd	29	−1·06	0·30			−3·68	**0·001**	1·28	0·21			−1·41	0·17	−1·15	0·26	−1·08	0·29	0·84	0·41		
		1st–2nd	29	−0·97	0·34			−0·42	0·68	0·27	0·79			−0·58	0·57	1·39	0·18	−0·05	0·96	−1·15	0·26		
		2nd–3rd	29	−0·09	0·93			−3·15	**0·004**	0·91	0·37			−0·81	0·43	−2·24	**0·03**	−0·92	0·36	1·79	0·08		
	Reindeer‐grazed sites	1st–3rd	27			−2·70	**0·01**					−5·86	**<0·001**									−2·75	**0·01**
		1st–2nd	27			0·02	0·99					0·00	1·00									−0·08	0·94
		2nd–3rd	27			−2·60	**0·02**					−5·74	**<0·001**									−2·63	**0·01**
	Fulufjället	1st–3rd	27			0·02	0·98					−0·92	0·37									0·46	0·65
		1st–2nd	27			−2·24	**0·03**					0·30	0·77									−1·05	0·30
		2nd–3rd	27			2·16	**0·04**					−1·19	0·24									1·48	0·15
Birch forest	Långfjället + Fulufjället	1st–3rd	17	0·00	1·00			−1·08	0·30					−1·87	0·08							−6·80	**<0·001**
		1st–2nd	17	−1·77	0·09			1·69	0·11					−0·03	0·98							−2·33	**0·03**
		2nd–3rd	17	1·54	0·14			−2·50	**0·02**					−1·64	0·12							−3·93	**0·001**
	Långfjället	1st–3rd	15			−0·87	0·40					−1·81	0·09			−2·58	**0·02**	−4·91	**<0·001**	3·41	**0·004**		
		1st–2nd	15			−1·31	0·21					1·54	0·14			−0·44	0·66	1·51	0·15	0·59	0·57		
		2nd–3rd	15			0·41	0·69					−3·18	**0·01**			−2·03	0·06	−6·00	**<0·001**	2·57	**0·02**		
	Fulufjället	1st–3rd	15			1·33	0·20					−7·27	**<0·001**			−6·14	**<0·001**	−0·64	0·53	0·40	0·69		
		1st–2nd	15			−3·60	**0·003**					−1·36	0·20			−5·22	0·00	1·44	0·17	1·64	0·12		
		2nd–3rd	15			4·60	**<0·001**					−5·61	**<0·001**			−0·87	0·40	−1·95	0·07	−1·13	0·28		
	Pulsuvuo.	1st–3rd	13			−2·17	0·05	−0·11	0·91			−8·85	**<0·001**	−2·07	0·06	−0·18	0·86	−0·57	0·58	2·24	**0·04**	0·31	0·76
		1st–2nd	13			−2·86	**0·01**	−0·75	0·47			−0·28	0·79	−0·58	0·58	−0·64	0·53	3·14	**0·01**	0·73	0·48	3·26	**0·01**
		2nd–3rd	13			0·28	0·78	0·55	0·59			−7·70	**<0·001**	−1·41	0·18	0·40	0·70	−3·21	**0·01**	1·31	0·21	−2·55	**0·02**

In terms of relative abundance, the shrub expanse at the birch forest sites occurred primarily at the expense of mosses despite the total increase of both groups (Fig. 4b).

### Field layer heights

Mixed effects models showed no significant differences in plant height between exclosures and ambient plots for any of the functional groups.

### Shrub layer

Five species of shrubs and saplings were recorded in the shrub layer inventories; *B. nana*,* S. glauca*,* B. pubescens* ssp. *czerepanovii*,* Juniperus communis* L. and *P. sylvestris*. *Juniperus communis* and *P. sylvestris* were excluded from the analyses as we could find no record of reindeer browsing these species in the literature. *Salix glauca* was only found at Ritsem, where it constituted less than 10% of the total shrub cover, and a total of four *B. pubescens* saplings were recorded at Fulufjället and Långfjället (tallest height 47 cm). *Betula nana*, in other words, made up the great majority of the tall shrub cover.

**Figure 5:**
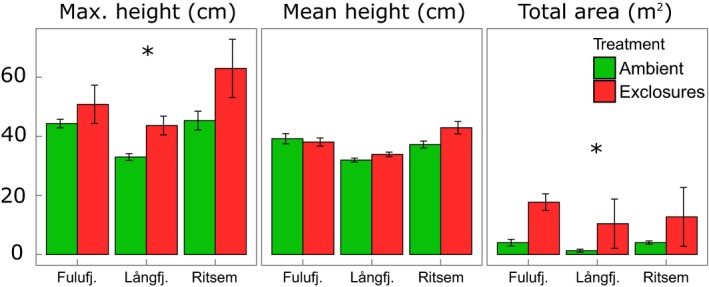
Height of tallest shrub, mean shrub height and mean area of shrub layer in the plots. Shrub layer is defined as shrubs >30 cm. Asterisks denote significant differences between ambient plots and exclosures at the 5% level. [Colour figure can be viewed at wileyonlinelibrary.com]

Tallest shrub height (d.f. = 1,10, *F* = 4·98, *P* = 0·05) and shrub area (d.f.  = 1,10, *F* = 6·70, *P *=* *0·03) were significantly greater in exclosures than in ambient plots, but mean shrub height was not (Fig. 5). Tallest shrub height and mean shrub height differed significantly between sites, an effect that *post hoc* tests showed to be driven mainly by Långfjället and not by reindeer‐free Fulufjället (Fulufjället did not differ significantly from Ritsem for either tallest or mean shrub height, whereas Långfjället did).

### Diversity

Field layer diversity showed no significant treatment effects or changes over time apart from a significant decrease over time in the Berger–Parker index at the birch forest sites between 1995 and 1999 (d.f. = 17, *t*‐ratio = 2·13, *P* = 0·05) and a significant positive effect of reindeer exclosure at Pulsuvuoma in 1999 (d.f.  = 12, *t*‐ratio = −2·215, *P* = 0·05).

For the 2011 data, Simpson's index, the Berger–Parker index and species richness calculations were based on the full species set and included the new ambient plot in Pulsuvuoma. The Simpson and Berger–Parker indices were both higher in ambient plots at all sites except Långfjället heath, but no significant effects of reindeer exclosure or between reindeer‐grazed sites and Fulufjället were found.

### Temperature measurements

**Figure 6:**
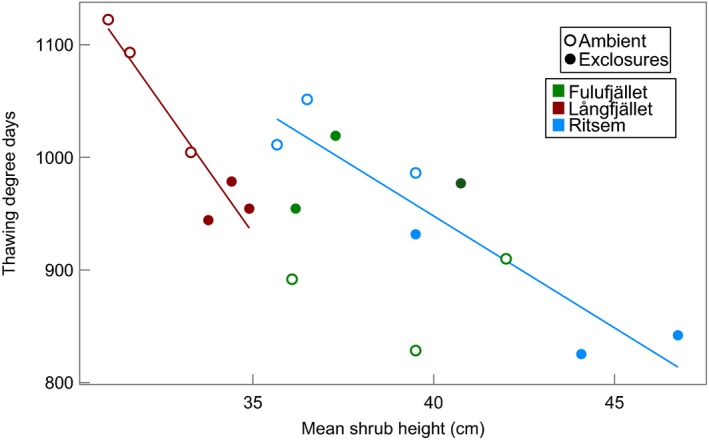
Relationship between mean shrub height and growing season TDD (calculated from soil temperatures). Regression line fitted where relationship is significant (Långfjället: d.f. = 1,4, *F* = 39·75, *P* = 0·003, *R* ^2^ = 0·89 and Ritsem: d.f. = 1,4, *F* = 21·28, *P *= 0·01, *R* ^2^ = 0·80). [Colour figure can be viewed at wileyonlinelibrary.com]

Mean July soil temperatures were higher in ambient plots than in exclosures at five of six sites, whereas mean January soil temperatures were higher in exclosures at four of six sites, including all the heath sites (Table 5). Neither July nor January temperatures differed statistically between treatments, but July temperatures were near significant (d.f. = 1,29, *F* = 3·98, *P* = 0·06). There was also a significant treatment effect on TDD sums (d.f. = 1,24, *F* = 4·63, *P *=* *0·04), which were higher in ambient plots at all but one site, Fulufjället heath. Furthermore, linear regression showed a significant negative correlation between mean tall shrub height and TDD at the reindeer‐grazed heath sites (d.f. = 1,4, *F* = 39·75, *P *=* *0·003 at Långfjället and d.f.  = 1,4, *F* = 21·28, *P *=* *0·01 at Ritsem, Fig. 6).

**Table 5:** Mean January and July soil temperatures and thawing degree day sums calculated from soil temperatures between 15 May and 15 September. July temperatures are from 2012 for all sites except Fulufjället birch forest (from 2013) and Ritsem (from 2014). Mean January temperatures are from 2013 for all sites except Ritsem (from 2014). There was a significant treatment effect on TDD, but not on July and January temperatures. Amb., ambient plots; Excl., exclosures

	Shrub heath						Mountain birch forest					
	Fulufjället		Långfjället		Ritsem		Fulufjället		Långfjället		Pulsuvuoma	
	Amb.	Excl.	Amb.	Excl.	Amb.	Excl.	Amb.	Excl.	Amb.	Excl.	Amb.	Excl.
July soil	9·1 ± 0·2	9·7 ± 0·2	10·3± 0·3	9·2 ± 0·1	13·9 ± 0·2	13·0 ± 0·4	10·8 ± 0·2	10·5 ± 0·6	10·0 ± 0·1	9·3 ± 0·4	7·3 ± 0·8	6·4 ± 1·2
Jan soil	−0·7 ± 0·1	−0·1 ± 0·1	−1·3 ± 0·1	−0·6 ± 0·2	−1·7 ± 0·6	−1·5 ± 0·3	0·8 ± 0·1	0·4 ± 0·1	0·0 ± 0·0	0·1 ± 0·2	−0·4 ± 0·6	−1·5 ± 0·7
TDD^a^	842 ± 7	927 ± 21	1009 ± 38	890 ± 8	973 ± 18	836 ± 27			1011 ± 28	936 ± 43	642 ± 53	570 ± 100

a *Significant difference between ambient and fenced plots (d.f. = 1,24, *F* = 4·63, *P* = 0·04).

**Figure 7:**
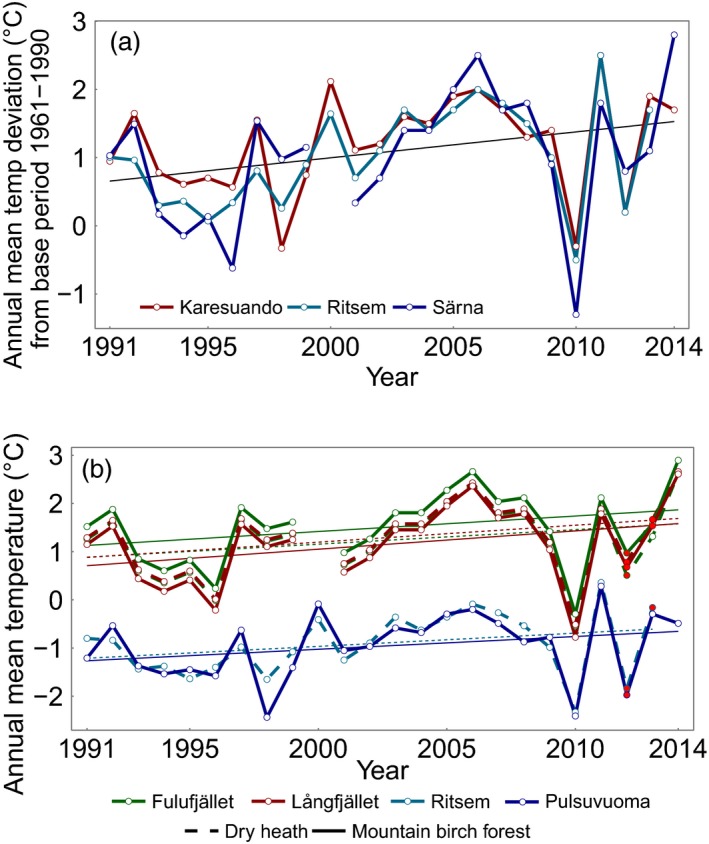
(a) Annual mean temperature deviation (°C) from the meterological base period of 1961–1990 at the nearest active SMHI meterological stations to our sites. Karesuando is the closest station to the Pulsuvuoma site, Ritsem to the Ritsem site and Särna to the Långfjället and Fulufjället sites. (b) Annual mean temperature at the study sites since the start of the current meteorological base period. Observed temperatures are indicated with red dots, remaining years have been extrapolated using SMHI data from the closest meteorological stations. [Colour figure can be viewed at wileyonlinelibrary.com]

Mean air temperatures from the closest SMHI stations (Karesuando, Ritsem and Särna) showed that between 1991 and 2014, all but two of the 24 years were warmer than the meterological base period mean of 1961–1990 in Karesuando and Särna. In Ritsem, all but 1 year was warmer (Fig. 7a). The mean temperature at the SMHI stations was 1·2 °C (Karesuando), 1·1 °C (Ritsem) and 1·2 °C (Särna) higher between 1991 and 2014 compared to 1961–1990 (SMHI 2017). The recorded (in red) and extrapolated temperatures from our site loggers are shown in Fig. 7b.

## Discussion

This study shows that between 1995 and 2011, the cover of evergreen dwarf shrubs increased dramatically across the Fennoscandian mountains, both at the heath and the birch forest sites (from 23% to 47% averaged over all sites and treatments). Deciduous shrub cover also increased in the field layer, although the increase was not as large and consistent (5–7% and 5–8%, for dwarf and tall shrubs respectively, averaged over all sites and treatments). In the shrub layer, however, deciduous shrubs were significantly greater and taller in exclosures than in ambient plots. Hence, earlier findings that large herbivores can inhibit the expansion of tall deciduous shrubs (Post & Pedersen 2008; Olofsson *et al*. 2009) are supported by our results. The overriding vegetation shift across our sites, however, was the striking increase in evergreen dwarf shrubs which has not been influenced by grazing.

Though our results seem to be at odds with recent findings that climate sensitivity is greater for tall rather than low‐statured shrubs (Myers‐Smith *et al*. 2015), models have predicted that increasing summer temperatures will cause an expanse in range of *C. vulgaris* (Crawford & Jeffree 2007), and both a range extension (Alsos *et al*. 2007) and an altitudinal advancement (Klanderud & Birks 2003) of *E. nigrum* have been noted in the past decade. Both these dominant species in our study have previously been shown to respond positively to experimental warming (Buizer *et al*. 2012; Kaarlejärvi *et al*. 2012; Kaarlejärvi, Hoset & Olofsson 2015). Similar increases of *E. nigrum* above the tree line were found in a long‐term monitoring study in northern Sweden (Wilson & Nilsson 2009). Clearly, the ability of *E. nigrum* to respond to warmer temperatures in combination with its allelopathic qualities and ability to immobilize soil nutrients (Nilsson 1994) have helped it to consolidate its dominance at our nutrient‐poor sites. Nonetheless, considering that stress‐tolerant species such as *E. nigrum* are expected to be slow to respond to environmental change and altered competition (Grime 2001), the scale of the evergreen dwarf shrub advancement in our study was surprising.

There could be several possible reasons for the observed expansion of evergreens. Though tall deciduous shrubs, such as *B. nana*, have been shown to be among the most responsive tundra species to increasing temperatures and have often been found to increase at the expense of evergreen shrubs (Chapin *et al*. 1995; Bret‐Harte *et al*. 2001), it is conceivable that, under moderate grazing pressure, less palatable evergreens could be given a competitive advantage despite their semi‐prostrate growth form (Christie *et al*. 2015; Ylänne, Stark & Tolvanen 2015). In our study, however, the advancement of evergreen dwarf shrubs in both ambient plots and exclosures suggests that this was not a result of altered competitive interactions between shrub species due to a release from grazing. Previous studies in Fennoscandian reindeer herding districts, too, have found that evergreen dwarf shrubs maintain a similar dominance in low‐productive habitats at both high and low reindeer densities (Bråthen *et al*. 2007), indicating that reindeer influence on these interactions are limited. Furthermore, many studies have shown that lemmings and voles can have a considerable impact on ericoid shrubs (Dahlgren *et al*. 2009; Olofsson, Tømmervik & Callaghan 2012; Olofsson *et al*. 2014), and that they may have a more marked effect on vegetation than reindeer (Olofsson *et al*. 2004). Although we do not have data on lemming and vole cycles from our sites, there is evidence that these have become less frequent in the Scandes over the past 15 years (Kausrud *et al*. 2008; Ims, Yoccoz & Killengreen 2011). Hence, a decreased long‐term grazing pressure from rodents could be an important contributing factor in the observed expansion of evergreen dwarf shrubs.

It is also possible that it is the increasingly mild winters (SMHI 2012), rather than warmer summers, during our study period that have had the largest impact on the vegetation. The southern and northern oceanic parts of the Scandes are characterized by mild winters and are dominated by ericoid shrub heaths, whereas the colder more continental parts are to a greater extent distinguished by dwarf birch heaths (Oksanen & Virtanen 1995; Virtanen *et al*. 2016), and an increase in winter temperatures at our ericoid‐dominated sites may have favoured evergreen dwarf shrubs like *C. vulgaris*, which have a relatively low tolerance to freezing (Körner 2003). However, even the most continental birch forest site, where there is no *C. vulgaris*, Pulsuvuoma, showed a dramatic increase in evergreen dwarf shrubs. Furthermore, milder winters and an increased frequency of extreme weather events may also increase the risk of frost damage to vegetation (arctic browning) as it is exposed to temporary high temperatures, that reduce frost‐tolerance, before temperatures again drop, and evergreen shrubs may be particularly susceptible to this (Bokhorst *et al*. 2008, 2009; Phoenix & Bjerke 2016). Taken together, evidence suggests that multiple factors, to varying degrees, have influenced the observed increase in evergreen shrubs.

Lichens, mosses and deciduous prostrate dwarf shrubs showed significant treatment effects at the heath sites in 2011. Lichens and *S. herbacea*, which was the dominant deciduous prostrate dwarf shrub, are important sources of food for reindeer (Eriksson, Niva & Caruso 2007) and it follows that they were more abundant in exclosures. However, at Långfjället, lichen cover had decreased in ambient plots, whereas in exclosures it remained at the same level, while at Ritsem, the difference was mainly caused by an increase in exclosures. This could be explained by the fact that grazing has intensified in the area of our Långfjället study site in the last 10 years (J. Jonsson, Idre Sami village, pers. comm. 2014) even though the number of reindeer in total has stayed roughly constant. However, in Fulufjället, where there have been no reindeer for more than a 100 years, lichen cover had also decreased, suggesting that the reason could be more complex than just a change in grazing pressure. Lichens could be negatively affected by the increase of shrubs, through shading (Cornelissen *et al*. 2001) and increased litter deposition (Chapin *et al*. 1995), and therefore we believe that competition with shrubs prevented an increase in lichens in exclosures in Långfjället, even though grazing ceased, whereas in ambient plots, lichens decreased as a result of the combination of increased competition and grazing. At the northerly site of Ritsem, even though deciduous shrubs had increased in exclosures, there too total shrub cover and the competition with evergreens were lower, allowing lichens to expand in response to a release from grazing pressure.

Mosses on the other hand, which are also sensitive to shading but less preferred as reindeer fodder (Danell *et al*. 1994), were more abundant in ambient plots at both Långfjället heath and Ritsem, whereas at the birch forest sites, there was no treatment effect but a significant increase over time. At Ritsem, there was a general decline in moss cover irrespective of treatment. This may in part have been caused by lemming activity, as 2011, the year of our inventories, saw a major lemming peak (Kaarlejärvi, Hoset & Olofsson 2015), and there were signs of intense grazing, particularly on the moss cover, in both exclosures and ambient plots at Ritsem. Even though a long‐term decrease in grazing pressure may have aided the expanse of ericoid shrubs, as previously discussed, a peak around the time of our inventories may be the reason for the overall decline in moss cover at Ritsem.

Diversity in the form of evenness, though not significant, tended to be higher in ambient plots than in exclosures, though we saw no changes in species richness or diversity in the field layer over the years. Earlier studies have found that increased shrub cover negatively affects species richness (Pajunen, Oksanen & Virtanen 2011) but we could see no connection between shrub cover increase and diversity in our study. Also, previous experiments have found that experimental warming decreases moss and lichen species richness (Jägerbrand *et al*. 2006; Dawes *et al*. 2011) but due to the difficulties with identifying lichen and bryophyte species in the field and uncertainties about possible inconsistencies in the inventories between years, we dare not make any deductions about changes in bottom layer biodiversity over time. A more detailed study of lichen and bryophyte community change over time is needed to draw such conclusions, but we cautiously note that out of the heath sites, the lowest diversity is found at Fulufjället, where there has been no grazing for the past century. Hence, grazing history may play a large part in ecosystem responses to increasing temperatures, especially at unproductive dry heath sites such as these, which suggests that more than 16 years may be needed to give a clearer picture of the effects of plant–herbivore interplay in a changing climate.

Soil temperatures were generally lower in exclosures in summer and higher in winter, which is consistent with earlier findings that shrub cover has contrasting effects on soil temperatures in summer and winter (Sturm *et al*. 2001; Blok *et al*. 2010; Myers‐Smith & Hik 2013). Growing season TDD were significantly higher in ambient plots and there was a significant negative correlation between mean shrub height and TDD at the reindeer‐grazed heath sites, which is most likely an effect of increased shading. Although rodents and hares, which have been found to exert a strong browsing pressure on *B. nana* (Vowles *et al*. 2016), were not prevented from accessing the exclosures, the exclusion of large herbivores had a significant effect on both shrub height and cover of tall shrubs at our sites. This suggests that even though the effect on ericoid shrub species was minor, reindeer browsing of tall, deciduous shrubs, could still impact key ecosystem functions such as shading, with knock‐on effects for soil temperature and nutrient cycling. Furthermore, in winter, tall shrub canopies can raise soil temperatures through amplified snow trapping (Sturm *et al*. 2001; Myers‐Smith & Hik 2013). Higher winter soil temperatures have, in turn, been found to raise over‐winter nitrogen mineralization rates and thereby alter the timing and amount of plant‐available nitrogen in tundra ecosystems (Schimel, Bilbrough & Welker 2004). However, even though January soil temperatures tended to be higher in exclosures, the link between grazing and winter soil temperatures at our sites is inconclusive, as we could see no correlation between shrub cover or height and January soil temperatures. It is also possible that the fences themselves contributed to an increased snow depth, but we could see no differences in snow depth at the two sites that we visited regularly during winter, Långfjället heath and birch forest (T. Vowles, unpublished data).

In conclusion, we have found that the exclusion of large herbivores amplified deciduous tall shrub expansion, as hypothesized. However, contrary to many previous studies, the major vegetation change at our sites was a dramatic increase in the already dominant evergreen dwarf shrubs, *E. nigrum* and *C. vulgaris* in particular, which were not influenced by grazing. The associated effects of an increased tall shrub cover, such as an accelerated nutrient cycling due to a deeper snow cover, have been given a great deal of attention, but the effects of an increase in evergreen shrubs and more recalcitrant plant litter have not been discussed to the same extent. As these effects may to some degree counteract each other, more research is needed into the outcome of competitive shrub interactions on ecosystem processes such as C‐cycling, and what part grazing may play in regulating these interactions.

## Authors’ contributions

T.V., U.M., T.H., L.K. and R.G.B. conceived the ideas and designed methodology; T.V., U.M. and R.G.B. collected the data; T.V. and R.G.B. analysed the data; T.V. led the writing of the manuscript with the help of B.G. and R.G.B. All authors contributed critically to the drafts and gave final approval for publication.

## Data accessibility

Data from this paper can be accessed through Environment Climate Data Sweden (ECDS) https://doi.org/10.5879/ECDS/2017-01-29.1/0 (Vowles *et al*. 2017).

## Supporting information


**Table S1.** Site coordinates.

**Table S2.** Full data on individual species cover.
