# Supplementary material for: Expansion of deciduous tall shrubs but not evergreen dwarf shrubs inhibited by reindeer in Scandes mountain range
Source: J Ecol. 2017 Mar 16;105(6):1547–61. doi: 10.1111/1365-2745.12753 (PMC5697633; doi:10.1111/1365-2745.12753)
Supplement: Supplementary file 1 — Table S1. Site coordinates. [file JEC-105-1547-s001.pdf]

	Site	Plot	Treatment	N	E
Shrub heath	Fulufjället	1	Exclosure	N 61°38'08.1"	E 12°38'18.9"
		2	Exclosure	N 61°38'08.3"	E 12°38'23.5"
		3	Exclosure	N 61°38'09.4"	E 12°38'17.6"
		4	Ambient	N 61°38'07.5"	E 12°38'15.0"
		5	Ambient	N 61°38'05.2"	E 12°38'13.1"
		6	Ambient	N 61°38'10.3"	E 12°38'12.3"
	Långfjället	1	Ambient	N 62°06'49.8"	E 12°16'20.1"
		2	Exclosure	N 62°06'52.7"	E 12°16'13.7"
		3	Ambient	N 62°06'56.9"	E 12°16'11.9"
		4	Ambient	N 62°06'51.2"	E 12°16'31.5"
		5	Exclosure	N 62°06'54.3"	E 12°16'28.2"
		6	Exclosure	N 62°06'54.3"	E 12°16'20.5"
	Ritsem	1	Exclosure	N 67°46'27.2"	E 17°32'06.6"
		2	Ambient	N 67°46'23.0"	E 17°32'10.0"
		3	Exclosure	N 67°46'25.2"	E 17°32'11.4"
		4	Ambient	N 67°46'24.1"	E 17°32'07.8"
		5	Exclosure	N 67°46'18.9"	E 17°32'01.4"
		6	Ambient	N 67°46'23.0"	E 17°32'10.1"
Birch forest	Fulufjället	1	Exclosure	N 61°38'42.2"	E 12°35'24.5"
		2	Exclosure	N 61°38'43.4"	E 12°35'22.8"
		3	Exclosure	N 61°38'42.0"	E 12°35'20.6"
		4	Ambient	N 61°38'44.8"	E 12°35'32.5"
		5	Ambient	N 61°38'39.0"	E 12°35'18.8"
		6	Ambient	N 61°38'37.9"	E 12°35'24.9"
	Långfjället	1	Exclosure	N 62°03'55.5"	E 12°14'45.9"
		2	Ambient	N 62°03'55.6"	E 12°14'42.0"
		3	Ambient	N 62°04'01.6"	E 12°14'41.3"
		4	Exclosure	N 62°04'03.2"	E 12°14'39.0"
		5	Exclosure	N 62°03'57.8"	E 12°14'59.8"
		6	Ambient	N 62°03'55.5"	E 12°15'07.2"
	Pulsuvuoma	1	Exclosure	N 68°20'13.2"	E 21°19'15.6"
		2	Exclosure	N 68°20'13.9"	E 21°19'07.7"
		3	Ambient	N 68°20'14.0"	E 21°19'10.5"
		4	Ambient	N 68°20'13.0"	E 21°19'12.2"
		5	Exclosure	N 68°20'09.8"	E 21°19'19.8"
		6*	Ambient	N 68°20'09.2"	E 21°19'16.5"

Coordinate system WGS84 *New plot
