# Supplementary material for: Expansion of deciduous tall shrubs but not evergreen dwarf shrubs inhibited by reindeer in Scandes mountain range
Source: J Ecol. 2017 Mar 16;105(6):1547–61. doi: 10.1111/1365-2745.12753 (PMC5697633; doi:10.1111/1365-2745.12753)
Supplement: Supplementary file 2 — Table S2. Full data on individual species cover. [file JEC-105-1547-s002.pdf]

Table S1 Full data on individual species cover. Cover data is given as means of visually estimated percentage covers. 0.00 in the table denotes a value <0.05.

			Dec.prostrate dwarf		Dec.semi-pr. dwarf		Deciduous Tall				
**Shrub heath**											
Site	Year	Treatment	Sali.herb	Arct.alpi	Rubu.cham	Vacc.myrt	Betu.nana	Betu.pube	Sali.glau	Sorb.aucu	Vacc.ulig
Fulufj.	1995	Ambient		0.05		0.67	1.48				
		Fence				1.34	5.20				
	1999	Ambient		0.07		2.54	3.27				
		Fence		0.10		3.01	7.22				
	2011	Ambient		0.02		0.77	7.52	0.00			
		Fence		0.15		0.93	8.92				
Långfj.	1995	Ambient		0.01		0.76	9.44	0.00			0.02
		Fence		0.05		0.87	9.89				0.02
	1998	Ambient				1.46	7.95				0.00
		Fence		0.03		0.83	16.54				0.02
	2011	Ambient				1.64	10.64	0.05			0.03
		Fence		0.10		2.40	19.32	0.00			0.00
Ritsem	1995	Ambient	6.42		0.04	4.09	0.29				
		Fence	9.16		0.05	1.84	2.43		0.12		
	1997	Ambient	10.72		0.00	2.37	0.31		0.00		0.00
		Fence	13.93		0.03	1.51	1.74		0.05		0.08
	2011	Ambient	14.00			9.10	2.01		0.27		
		Fence	25.70		0.17	6.40	8.62		0.83		0.42
**Mountain birch forest**											
Fulufj.	1995	Ambient				7.42	1.21	0.42			8.98
		Fence				11.44	1.00	0.02			7.52
	1999	Ambient				18.79	0.75	0.13			6.40
		Fence		0.02		18.60	1.33	0.23			10.73
	2011	Ambient				6.09	1.27	1.70			6.97
		Fence		0.00		5.19	0.32	0.05			9.39
Långfj.	1995	Ambient				11.43	0.07	0.11		0.03	4.17
		Fence				7.29	0.72	0.04			3.11
	1998	Ambient		0.04		13.93	1.40	0.07		0.02	2.59
		Fence				13.83	0.35	0.15			2.07
	2011	Ambient				12.72	0.29	0.05		0.00	6.82
		Fence				12.64	1.88	0.21	0.00	0.00	4.19
Tavva.	1995	Ambient				5.66	0.00	0.22			1.14
		Fence	0.00		0.00	3.92	0.04	0.08			0.64
	1999	Ambient				15.65	0.10	0.34			1.89
		Fence				12.59	0.04	0.35			2.12
	2011	Ambient				12.60	0.00	0.10			1.03
		Fence			0.00	16.88		0.10			2.39

If a species covered less than 1% of a subplot it was given the cover 0.1

			Ever.prostrate dwarf		Evergr. Semi-prostrate dwarf					Evergr. tall	
**Shrub heath**											
Site	Year	Treatment	Cass.hypn	Lois.proc	Andr.poli	Call.vulg	Empe.herm	Phyl.caer	Vacc.viti	Juni.comm	Pinu.sylv
Fulufj.	1995	Ambient				5.82	23.12	0.39	1.69	1.27	
		Fence				16.12	13.75	1.08	2.62		
	1999	Ambient				5.87	18.02	0.65	3.64	0.35	
		Fence				10.24	20.37	0.93	3.33		
	2011	Ambient				13.75	24.64	0.50	2.88	1.02	
		Fence				28.08	18.30	0.49	3.64		
Långfj.	1995	Ambient		0.05	0.03	19.52	21.62	0.56	2.66		
		Fence		0.92		7.59	24.89	0.34	3.05		
	1998	Ambient		0.33	0.03	17.88	25.02	1.63	2.51		
		Fence		0.32		8.36	26.18	1.85	3.02		
	2011	Ambient		0.04		37.97	48.67	1.14	5.36		
		Fence		0.25		22.72	52.80	1.22	7.24		
Ritsem	1995	Ambient	1.44	0.07			3.58	0.24	0.68		
		Fence	3.57	0.17			5.54	0.66	0.34		
	1997	Ambient	0.74	0.00			2.97	0.14	0.99		
		Fence	0.29	0.10			4.53	0.49	0.16		
	2011	Ambient	1.02	0.50			10.37	0.29	2.12		
		Fence	0.54	0.23			7.87	0.50	2.47		
**Mountain birch forest**											
Fulufj.	1995	Ambient				3.20	13.54		1.63	0.75	
		Fence			0.00	5.09	11.69		2.76	0.40	
	1999	Ambient				3.02	17.12		3.65	0.10	
		Fence			0.06	4.18	14.20		3.07	0.14	
	2011	Ambient				7.90	33.58		6.44	2.12	
		Fence			0.03	11.35	28.53		4.02	1.02	
Långfj.	1995	Ambient				3.83	19.82		8.50	0.30	
		Fence				6.40	19.20		7.82	0.12	
	1998	Ambient				5.50	18.00		3.92	1.05	
		Fence				8.69	23.22		4.47	0.74	0.00
	2011	Ambient				5.80	26.29	0.00	9.52	1.45	0.00
		Fence				11.92	32.10		9.20	2.27	0.00
Tavva.	1995	Ambient					3.22		3.28	1.89	
		Fence					4.52		2.65	1.67	
	1999	Ambient					4.03		3.10	1.97	
		Fence					4.39		4.32	2.28	
	2011	Ambient					18.92		21.42	6.29	
		Fence					22.45		19.75	3.97	

			Forbs												
**Shrub heath**															
Site	Year	Treatment	Ante.alpi	Ante.dioi	Bart.alpi	Bist.vivi	Cera.font	Diph.alpi	Diph.comp	Equi.arve	Equi.sylv	Gent.niva	Gnap.supi	Hier.alpi	Hupe.sela
Fulufj.	1995	Ambient													
		Fence													
	1999	Ambient													
		Fence													
	2011	Ambient												0.00	
		Fence													
Långfj.	1995	Ambient						0.00	0.00						
		Fence													
	1998	Ambient						0.00							
		Fence													
	2011	Ambient						0.03							
		Fence													
Ritsem	1995	Ambient		0.00		1.62		0.29		0.02		0.00	0.35	0.81	0.03
		Fence				1.71		0.05					0.29	1.04	0.00
	1997	Ambient		0.03		1.46		0.09						0.92	0.01
		Fence		0.02		0.48	0.00						0.01	0.87	0.01
	2011	Ambient	0.09			1.37		0.15		0.02		0.00	0.34	1.00	0.01
		Fence	0.09			1.42		0.08					0.03	1.69	0.09
**Mountain birch forest**															
Fulufj.	1995	Ambient												0.00	
		Fence													
	1999	Ambient													
		Fence													
	2011	Ambient													
		Fence													
Långfj.	1995	Ambient													
		Fence													
	1998	Ambient													
		Fence													
	2011	Ambient													
		Fence							0.00						
Tavva.	1995	Ambient						0.01							
		Fence			0.22			0.02							
	1999	Ambient						0.00							0.00
		Fence						0.01	0.02		0.02				
	2011	Ambient						0.02							
		Fence												0.02	

Forbs continued

Shrub heath

Site	Year	Treatment	Linn.bore	Lyco.anno	Lyco.clav	Mela.prat	Other.forbs	Pedi.lapp	Peta.frig	Pyro.mino	Ranu.acri	Ranu.niva	Rume.acet	Rhod.rose	Sela.sela
Fulufj.	1995	Ambient													
		Fence													
	1999	Ambient													
		Fence													
	2011	Ambient													
		Fence													
Långfj.	1995	Ambient													
		Fence													
	1998	Ambient													
		Fence													
	2011	Ambient													
		Fence													
Ritsem	1995	Ambient					3.15	0.01	0.00		0.00	0.01	0.04	0.09	
		Fence			0.00		3.29	0.10				0.00	0.07		
	1997	Ambient		0.05			0.00	0.02				0.00	0.10	0.09	
		Fence		0.00			0.00	0.05					0.12		
	2011	Ambient		0.00				0.09	0.12	0.03	0.12	0.03	0.04	0.41	0.00
		Fence			0.02			0.18				0.00	0.06		
**Mountain birch forest**															
Fulufj.	1995	Ambient				0.26	0.00								
		Fence				0.73									
	1999	Ambient				2.30									
		Fence				2.56									
	2011	Ambient		0.17		2.51									
		Fence	0.33			1.16									
Långfj.	1995	Ambient	0.23	0.02		0.92									
		Fence		0.25		0.80									
	1998	Ambient	0.67	0.00		1.06									
		Fence	0.14	0.19		0.93									
	2011	Ambient	0.39	0.05		1.40									
		Fence	0.15	0.72		0.36									
Tavva.	1995	Ambient	0.36	0.41				0.09							
		Fence	0.32	0.05				0.25							
	1999	Ambient	0.46	0.00	0.28			0.03							
		Fence	1.09	0.05	0.32			0.04							
	2011	Ambient	1.08	0.00				0.01							
		Fence	0.78	0.43				0.03							

			Forbs continued					
**Shrub heath**								
Site	Year	Treatment	Sibb.proc	Soli.virg	Tara.sp	Trie.euro	Vero.alpi	Viol.bifl
Fulufj.	1995	Ambient				0.00		
		Fence						
	1999	Ambient						
		Fence				0.03		
	2011	Ambient						
		Fence				0.04		
Långfj.	1995	Ambient						
		Fence				0.00		
	1998	Ambient				0.00		
		Fence				0.00		
	2011	Ambient				0.08		
		Fence				0.00		
Ritsem	1995	Ambient	0.05	1.22	0.02	0.50	0.00	0.03
		Fence	0.13	0.22		0.52		
	1997	Ambient	0.06	1.18	0.05	1.10	0.00	0.01
		Fence	0.07	0.44		0.42		
	2011	Ambient	0.20	3.67	0.04	0.61	0.01	0.23
		Fence	0.04	2.66	0.02	0.40		
**Mountain birch forest**								
Fulufj.	1995	Ambient		0.00		0.15		
		Fence		0.00		0.01		
	1999	Ambient		0.02		0.23		
		Fence		0.04		0.11		
	2011	Ambient		0.00		0.56		
		Fence		0.02		0.16		
Långfj.	1995	Ambient		0.02		0.24		
		Fence		0.11		0.30		
	1998	Ambient		0.01		0.28		
		Fence		0.05		0.31		
	2011	Ambient		0.22		0.67		
		Fence				0.55		
Tavva.	1995	Ambient		0.31		0.43		
		Fence		0.36		0.25		
	1999	Ambient		0.49		0.79		
		Fence		0.37		0.31		
	2011	Ambient		0.26		0.30		
		Fence		0.27		0.12		

			Graminoids												
**Shrub heath**															
Site	Year	Treatment	Agro.mert	Anth.odor	Cala.lapp	Cala.purp	Care.atra	Care.bige	Care.brun	Care.lach	Carex.sp	Desc.flex	Erio.vagi	Fest.ovin	Junc.trif
Fulufj.	1995	Ambient										1.85			
		Fence										0.93			
	1999	Ambient										1.38			
		Fence										0.80			
	2011	Ambient										2.07			
		Fence										4.06			
Långfj.	1995	Ambient						0.38				0.31		0.02	0.00
		Fence						0.74				0.53			
	1998	Ambient						0.56				0.23		0.02	
		Fence						0.62				0.25			0.02
	2011	Ambient						0.69				0.93			0.00
		Fence						0.67				1.09			0.05
Ritsem	1995	Ambient	1.20		4.58			5.38	1.38	1.55	0.00	0.34		0.20	0.17
		Fence	0.60	0.12	0.63			4.86	1.44	0.39	0.02	2.91		0.46	0.61
	1997	Ambient			0.76			3.83	0.69	1.44	0.17	0.33		0.04	0.03
		Fence	0.17	0.49	0.00		0.00	2.15	0.97	0.19	0.10	1.63		0.06	0.03
	2011	Ambient	0.13	0.19	5.90			3.72	0.16	0.11		0.40		0.00	0.02
		Fence	0.12	0.31	0.25			2.73	0.46			1.94			0.90
**Mountain birch forest**															
Fulufj.	1995	Ambient										15.00			
		Fence										8.30	0.32		
	1999	Ambient										7.89			
		Fence										5.49			
	2011	Ambient										13.15			
		Fence										12.10			
Långfj.	1995	Ambient										7.80			
		Fence										7.31			
	1998	Ambient										5.25		0.05	
		Fence										5.04		0.18	
	2011	Ambient										26.52			
		Fence										19.48			
Tavva.	1995	Ambient									0.02	14.80		0.27	
		Fence										9.52			
	1999	Ambient				0.00						5.64		0.38	
		Fence				0.00					0.00	7.19		0.17	
	2011	Ambient				0.14						12.47		0.96	
		Fence				0.06						6.80		0.06	

Site	Year	Treatment	Luzu.mult	Luzu.pilo	Luzu.spic	Moli.caer	Nard.stri	Other.gram	Phle.alpi	Poa.prat	Tris.spic	Vahl.atro
			Graminoids continued									
**Shrub heath**												
Fulufj.	1995	Ambient										
		Fence										
	1999	Ambient					0.13					
		Fence										
	2011	Ambient										
		Fence										
Långfj.	1995	Ambient										
		Fence										
	1998	Ambient										
		Fence										
	2011	Ambient										
		Fence										
Ritsem	1995	Ambient	0.07		0.00				0.00	0.01		0.01
		Fence	0.08		0.00				0.05	0.00		0.09
	1997	Ambient	0.07					7.02				
		Fence	0.07					4.63	0.15		0.00	0.00
	2011	Ambient							0.00			0.03
		Fence		0.00								0.07
**Mountain birch forest**												
Fulufj.	1995	Ambient		0.18			3.17	0.00				
		Fence		0.01		0.04	7.39					
	1999	Ambient		0.16			5.42					
		Fence		0.06		0.17	6.72					
	2011	Ambient		0.56			6.87					
		Fence		0.30		0.07	8.32					
Långfj.	1995	Ambient		0.41			0.38					
		Fence		0.35								
	1998	Ambient		0.18				0.00				
		Fence		0.08			0.05					
	2011	Ambient		0.99			0.35					
		Fence		0.35								
Tavva.	1995	Ambient						0.01				
		Fence										
	1999	Ambient										
		Fence										
	2011	Ambient										
		Fence										

Lichens continued

Site	Year	Treatment	Alec.nigr	Alec.ochr	Cetr.cucu	Cetr.eric	Cetr.isla	Cetr.niva	Clad.arbu	Clad.bell	Clad.cocc	Clad.cris	Clad.digi	Clad.fimb	Clad.grac
**Shrub heath**															
Fulufj.	1995	Ambient				1.48	9.40	2.66	18.95	0.22	0.27				0.39
		Fence				1.02	7.97	1.68	20.42	0.01	0.01				0.13
	1999	Ambient				1.03	11.87	0.09	19.45	0.33	0.05				1.21
		Fence				1.49	10.08	0.08	19.05	0.21	0.00			0.00	1.53
	2011	Ambient				1.00	6.97	0.33	19.34	0.20	0.21	0.45		0.01	0.01
		Fence				0.68	7.64	0.15	17.22	0.07	0.57	0.38			
Långfj.	1995	Ambient			0.12	0.91	1.28	1.81	5.82		0.01	0.08		0.04	0.91
		Fence			0.18	1.79	1.77	2.53	8.37	0.13	0.06	0.00		0.19	1.63
	1998	Ambient			0.28	3.39	2.73	2.08	10.97		0.02	0.08		0.04	0.91
		Fence			0.33	5.45	2.02	3.25	10.04	0.13	0.07	0.00		0.19	1.66
	2011	Ambient			0.14	2.19	0.62	0.53	10.72	0.01	0.09	0.02		0.04	1.12
		Fence			0.14	1.81	1.42	1.22	12.67	0.15	0.09	0.01		0.02	2.63
Ritsem	1995	Ambient		0.00		0.16	0.00		0.70	0.02					
		Fence	0.00	0.00		0.25	0.00		0.60	0.03					
	1997	Ambient				0.24	0.01	0.00	1.65	0.02	0.11				
		Fence				0.20	0.01		1.03	0.02	0.03			0.00	
	2011	Ambient				0.30	0.10	0.00	1.97	0.07	0.12	0.00		0.31	0.34
		Fence			0.00	1.11	0.98	0.01	5.14	0.18	0.23	0.08		0.36	2.26
**Mountain birch forest**															
Fulufj.	1995	Ambient					2.60		5.92	0.00	0.02				0.06
		Fence				0.00	4.10		6.16	0.00					
	1999	Ambient					2.91		3.52	0.00					0.14
		Fence					2.88		2.95	0.01					0.01
	2011	Ambient					3.63		5.05	0.00	0.02		0.05	0.08	0.12
		Fence					3.65		2.00		0.03			0.00	0.03
Långfj.	1995	Ambient				0.00	0.09		1.33						
		Fence				0.01	0.07	0.07	3.07						
	1998	Ambient					0.15		2.04	0.08	0.01	0.04		0.03	0.00
		Fence					0.15		3.35	0.02	0.00	0.06		0.05	
	2011	Ambient					0.08		0.73		0.01			0.01	0.04
		Fence				0.03	0.22		2.11	0.00	0.01			0.02	0.42
Tavva.	1995	Ambient					0.00		0.17			0.01		0.01	0.03
		Fence			0.00	0.00	0.00		0.34		0.00	0.03		0.03	0.04
	1999	Ambient					0.00		0.30	0.00				0.01	0.05
		Fence					0.02		0.79	0.00				0.01	0.16
	2011	Ambient							0.11	0.00	0.00			0.01	0.04
		Fence					0.00		0.19					0.01	0.04

			Lichens continued												
**Shrub heath**															
Site	Year	Treatment	Clad.pyxi	Clad.rang	Clad.sp	Clad.squa	Clad.stel	Clad.sulp	Clad.unci	Clad.corn	Dipl.scru	Icma.eric	Neph.arct	Ochr.frig	Otherlich
Fulufj.	1995	Ambient		1.27	0.77		22.94	0.19	0.30	0.56					1.92
		Fence		1.34	1.15		20.29	0.05	0.53	0.65		0.00			0.48
	1999	Ambient		0.00	0.95	0.14	22.80	0.20	0.20	0.09					0.09
		Fence		0.68	0.89	0.09	14.69	0.07	0.64	0.14					0.05
	2011	Ambient		1.29		0.01	21.79	0.09	0.12	0.04				0.00	0.25
		Fence		3.06			13.62	0.01		0.04		0.00		0.04	0.02
Långfj.	1995	Ambient		2.31	0.34	0.00	12.02	0.01	0.90	0.91					0.14
		Fence		3.22	0.55	0.01	9.57	0.03	2.00	1.52					0.38
	1998	Ambient		4.21	0.23	0.00	6.42	0.05	0.98	0.14					0.37
		Fence		3.35	0.36	0.01	7.01	0.02	3.38	0.30					1.86
	2011	Ambient		0.99		0.02	3.77	0.00	0.37	0.01					0.00
		Fence		4.43		0.03	8.31	0.00	1.09	0.02					0.01
Ritsem	1995	Ambient		0.01	2.71				0.16	0.11		0.12	2.38		3.15
		Fence		0.01	4.10				0.36	0.14	0.00	0.08	1.08		3.29
	1997	Ambient		0.01	1.89		0.00		0.40	0.23	0.00	0.28	1.06	0.18	3.90
		Fence		0.01	1.44				0.17	0.14	0.00	0.01	0.57	0.16	3.09
	2011	Ambient	0.09	0.01		0.07	0.00	0.02	0.65	0.01			3.49	1.13	0.01
		Fence	0.08	0.03		0.20	0.00	0.13	2.27	0.05			1.04	0.90	0.10
**Mountain birch forest**															
Fulufj.	1995	Ambient		3.42	0.14		1.65	0.00	0.14	0.22		0.00			0.19
		Fence		2.75	0.21		1.39	0.03	0.22	0.32		0.50			0.12
	1999	Ambient		2.28	0.08		0.80	0.06	0.07	0.06					
		Fence		1.44	0.15		1.87	0.03	0.02	0.10					
	2011	Ambient	0.02	3.69	0.00	0.00	0.37	0.00	0.00	0.20					
		Fence		0.87			0.29	0.00	0.02	0.03					0.01
Långfj.	1995	Ambient		1.12	0.25		0.26	0.01	0.16	0.37					0.03
		Fence		1.74	0.30		0.65	0.00	0.51	0.36					0.00
	1998	Ambient	0.00	1.14	0.08		0.06	0.04	0.17	0.14					0.02
		Fence		1.87	0.37		0.49	0.06	0.17	0.33			0.01		0.00
	2011	Ambient	0.00	0.52		0.00	0.00	0.01	0.01	0.02					
		Fence		1.10		0.01	0.64	0.04	0.14	0.00					
Tavva.	1995	Ambient		0.16	0.05		0.02	0.02	0.05	0.02			0.38		
		Fence		0.42	0.07		0.01	0.02	0.04	0.01			0.42		0.02
	1999	Ambient		0.21	0.06			0.01	0.06	0.01			0.14		0.02
		Fence		0.45	0.24		0.01	0.03	0.14	0.03		0.00	0.22		0.00
	2011	Ambient	0.00	0.04		0.00		0.00	0.01				0.07		0.00
		Fence		0.23			0.05	0.02	0.01				0.05		

Lichens continued

Site	Year	Treatment	Pelt.apht	Pelt.scab	Pelt.sp	Pert.dact	Solo.croc	Ster.spp	Tham.verm
**Shrub heath**									
Fulufj.	1995	Ambient							
		Fence						0.00	
	1999	Ambient							
		Fence							
	2011	Ambient							
		Fence							
Långfj.	1995	Ambient						0.00	
		Fence						0.44	
	1998	Ambient						0.02	
		Fence						0.14	
	2011	Ambient							
		Fence						0.04	
Ritsem	1995	Ambient					0.19	0.21	
		Fence					0.41	0.43	
	1997	Ambient					0.11	0.17	
		Fence	0.00				0.38	0.37	
	2011	Ambient	0.02	0.36		0.01	0.00	0.72	0.00
		Fence		0.26	0.01	0.02	0.03	2.41	0.42
**Mountain birch forest**									
Fulufj.	1995	Ambient							
		Fence							
	1999	Ambient							
		Fence							
	2011	Ambient	0.01						
		Fence							
Långfj.	1995	Ambient							
		Fence							
	1998	Ambient	0.00						
		Fence							
	2011	Ambient							
		Fence							
Tavva.	1995	Ambient	0.13						
		Fence	0.15					0.01	
	1999	Ambient	0.19					0.00	
		Fence	0.04					0.01	
	2011	Ambient	0.10						
		Fence	0.34						

			Mosses												
**Shrub heath**															
Site	Year	Treatment	Barb.lyco	Bart.ithy	Dicr.elon	Dicr.scop	Dicr.sp	Hylo.sple	Liverwort	Other.mos	Palu.squa	Plag.pili	Pleu.schr	Poly.alpi	Poly.comm
Fulufj.	1995	Ambient					0.12		0.00	0.02			0.61		
		Fence					0.30		0.07				1.31		
	1999	Ambient					0.26		0.10				0.39		
		Fence					0.69		0.23				1.07		
	2011	Ambient		0.04		0.05	0.00			0.01			3.11		
		Fence				0.05	0.22			0.01			2.65		
Långfj.	1995	Ambient					4.46		0.64	0.00			1.95		0.89
		Fence					4.19		0.56	0.00			0.84		1.16
	1998	Ambient					6.00		0.60	0.02			3.66		
		Fence					2.77		0.08	0.14			0.65		
	2011	Ambient	0.09	0.43	3.01	1.84		0.18					5.09		0.01
		Fence	0.10	0.00	0.14	0.85	0.00			0.04			2.36		0.01
Ritsem	1995	Ambient					24.87						0.12		
		Fence					25.49		0.00				0.02		
	1997	Ambient					41.62			0.45			0.13		
		Fence					35.25	0.04		1.06			0.12		
	2011	Ambient	7.66	0.06	9.35	10.43	0.08	0.95					0.09	0.65	
		Fence	7.08	0.04	5.49	9.97	0.07			0.35			0.02	0.37	
**Mountain birch forest**															
Fulufj.	1995	Ambient					3.18	0.64	2.00	0.64	0.07		14.37		
		Fence					5.06	0.67	0.65	0.02			9.32		
	1999	Ambient					4.07	1.49	2.14				11.54		
		Fence					5.09	1.44	0.96	0.02			7.79		
	2011	Ambient	8.55		0.32	3.85		1.37					25.75		0.00
		Fence	7.20	0.08	0.05	3.57	1.47	3.38		0.00		0.17	24.62		0.00
Långfj.	1995	Ambient					4.49	0.81	0.74	0.61			17.85		
		Fence					3.08	1.51	1.08	0.19			18.38		
	1998	Ambient					4.54	2.67	2.69	0.18			28.82		
		Fence					4.18	2.74	2.41	0.00			28.59		
	2011	Ambient	14.98		0.97	2.08	0.00	7.42				0.42	22.07		0.71
		Fence	6.18		0.21	1.25	0.05	5.15					35.65		0.14
Tavva.	1995	Ambient					2.99	5.84	0.39				57.30		5.28
		Fence					4.48	2.95	0.85	0.09	0.47		58.00		4.95
	1999	Ambient					1.91	4.82	0.70	0.10			50.20		
		Fence					2.25	2.87	1.74	0.00			42.97		
	2011	Ambient	1.03	0.01	0.07	0.98	0.01	6.87					57.52		6.09
		Fence	0.42	0.00		0.60		16.63					55.62		3.88

Mosses continued

Shrub heath

Site	Year	Treatment	Poly.juni	Poly.pili	Poly.sp	Poly.strict	Ptil.cili	Spha.sp
Fulufj.	1995	Ambient		0.09	0.00			
		Fence		0.01				
	1999	Ambient		0.06	0.12			
		Fence			0.02			
	2011	Ambient	0.01	0.02				
		Fence	0.00					
Långfj.	1995	Ambient		2.68			1.23	
		Fence		2.08			1.62	
	1998	Ambient		0.99	0.89		1.23	
		Fence		0.37	1.19		1.65	
	2011	Ambient	1.20				1.29	
		Fence	0.60	0.00			1.95	
Ritsem	1995	Ambient		15.77				
		Fence		15.52				
	1997	Ambient		5.94				0.02
		Fence		4.35				0.00
	2011	Ambient	3.08	0.21		1.60	0.00	
		Fence	2.28	0.20		0.59	0.00	
**Mountain birch forest**								
Fulufj.	1995	Ambient		0.02	0.07			
		Fence		0.19				0.50
	1999	Ambient			0.77			0.00
		Fence		0.55	0.02			0.42
	2011	Ambient					0.11	
		Fence	0.65		0.02			
Långfj.	1995	Ambient		0.07				
		Fence		0.13				
	1998	Ambient			0.28			
		Fence		0.02	0.06			
	2011	Ambient	0.03					
		Fence	0.05				0.00	
Tavva.	1995	Ambient		1.85				
		Fence		4.07				
	1999	Ambient	0.00		4.21		0.00	
		Fence	0.32		9.01			
	2011	Ambient	0.93		0.04		0.03	
		Fence	1.20				0.00	
